# Assessing the Clinical Efficacy of Dupilumab and Its Impact on the Quality of Life of Adult Atopic Dermatitis Patients: A Systematic Review

**DOI:** 10.7759/cureus.79762

**Published:** 2025-02-27

**Authors:** Mooroogiah Krissheeven, Sarvesh Nunkoo, Maheshwara Ramanah, Jared Robinson, Indrajit Banerjee

**Affiliations:** 1 Department of Pharmacology, Sir Seewoosagur Ramgoolam Medical College, Belle Rive, MUS; 2 Department of Surgery, Sir Seewoosagur Ramgoolam Medical College, Belle Rive, MUS

**Keywords:** atopic dermatitis (ad), dupilumab, inflammatory skin diseases, monoclonal antibodies (mabs), novel pharmacotherapy, skin disease/dermatology

## Abstract

Dupilumab, a monoclonal antibody, acts as a dual-action inhibitor that effectively suppresses interleukin-4 (IL-4) and interleukin-13 (IL-13) secretion, which plays an important role in type 2 inflammation. Atopic dermatitis (AD), also known as eczema, is a chronic inflammatory skin disorder that affects both adults and infants. The primary objective of this systematic review was to evaluate the clinical efficacy of dupilumab and the quality of life (QOL) of atopic dermatitis adult patients on dupilumab. Although adults experience a lower percentage of AD as compared to children globally, this disease should be addressed to improve the treatment of this condition in adults. The need for the study arises from the fact that there is a dearth of data regarding the efficacy of dupilumab in atopic dermatitis, particularly in the adult population. An extensive search was conducted using PubMed, Turning Research Into Practice (TRIP), and Cochrane Central Register of Controlled Trials (CENTRAL) and included studies published between 2018 and 2025 in accordance with Preferred Reporting Items for Systematic Reviews and Meta-Analyses (PRISMA) guidelines 2020. The Medical Subject Headings (MeSH) terms and Boolean operators used were "Dupilumab" OR "Dermatitis, Atopic". All randomized controlled trials (RCTs) were included in this systematic review. Nine full-text articles were ultimately considered, and critical appraisal was performed thereon. Dupilumab at a dose of 300 mg once every two weeks (q2w) and 300 mg once weekly (qw) were effective in treating atopic dermatitis by improving symptoms of pruritus, pain, and depression and preventing the spread of the disease. The quality of life (QOL) improved significantly, and the rate of atopic dermatitis hospitalization decreased after the use of dupilumab.

## Introduction and background

Background

Atopic dermatitis (AD), also known as eczema, is a chronic inflammatory skin disorder that affects both adults and infants. It is characterized by inflamed patches, redness, skin itchiness, pruritus, eczematous lesions, lichenification (thickening and hardening of the skin), and dry skin. It is often associated with other atopic conditions such as asthma, hay fever, allergic rhinitis, and food allergies. AD is thought to be caused by a combination of genetic and environmental factors [1].

Pathophysiology

The pathogenesis of atopic dermatitis primarily revolves around a type 2 immune-mediated reaction (an immune response primarily driven by T-helper 2 (Th2) cells in the inflammatory process), with interleukin-13 (IL-13) and interleukin-4 (IL-4). The cytokines play an important role in atopic dermatitis (AD) by driving inflammation and immune dysfunction. In AD, an overactive Th2 immune response prevails along with cytokines such as IL-4 and IL-13 promoting IgE production and skin barrier dysfunction and also a recruitment of inflammatory cells. Interleukin-5 (IL-5) contributes to eosinophil activation, while interleukin-17 (IL-17) and interferon-gamma (IFN-γ) from T-helper 1 (Th1) and T-helper 17 (Th17) cells drive chronic inflammation and further damage to the skin. This cytokine imbalance leads to skin inflammation, compromised barrier function of the skin, and itching, creating a cycle of flare-ups and chronic diseases [2].

Dupilumab (brand name: Dupixent), which is a biologic medication used to treat moderate to severe atopic dermatitis (AD), was approved by the US FDA in March 2017. Dupilumab is available in the market in two forms: single-dose pre-filled syringe with needle available in three strengths (300 mg/2 mL, 200 mg/1.14 mL, and 100 mg/0.67 mL (injection)) and single-dose pre-filled pen available in two strengths (300 mg/2 mL and 200 mg/1.14 mL (injection)) [3]. Dupilumab is a human monoclonal antibody that targets and inhibits two key proteins involved in the inflammatory process in AD: interleukin-4 (IL-4) and interleukin-13 (IL-13). Dupilumab is directed against the shared alpha subunit of the interleukin-4 receptors that blocks signaling from both interleukin-4 and interleukin-13, showing efficacy in patients with moderate to severe asthma and elevated eosinophil levels [2]. Dupilumab significantly decreased the serum levels of thymus and activation-regulated chemokine (TARC), which is a regulator of Th2-mediated immunity and a biomarker of AD disease. Moreover, dupilumab has been demonstrating sustained efficacy with improvements in signs and symptoms in adults along with quality of life (QOL) for up to five years in clinical studies. Dupilumab is much more advantageous over other drugs in terms of targeted action (specifically blocks IL-4 and IL-13 compared to a broad immune suppression with steroids). It has fewer side effects unlike systemic corticosteroids and also no skin thinning, unlike topical steroids. Dupilumab provides significant improvements in skin health and overall quality of life.

It is administered via subcutaneous injection, usually once every two weeks (300 mg), after an initial loading dose of 600 mg (two times 300 mg injection at two different sites). The following are the scoring systems that assess the clinical efficacy of dupilumab and the quality of life of patients that are used for investigation in clinical trials on atopic dermatitis: Eczema Area and Severity Index (EASI), Scoring Atopic Dermatitis (SCORAD), Dermatology Life Quality Index (DLQI), Numeric Rating Scale (NRS), and Hospital Anxiety and Depression Scale (HADS) [4,5].

Injection site reactions and side effects such as ophthalmic complications (e.g., dry eyes, conjunctivitis, blepharitis, keratitis, and ocular pruritus), progression of cutaneous T-cell lymphoma exacerbations, hyper-eosinophilia, head and neck dermatitis, the onset of psoriatic lesions, alopecia areata, and arthritis have been noted in patients on dupilumab [5-10].

Rationale of the study

The primary objective of this systematic review was to evaluate the clinical efficacy of dupilumab and the quality of life of atopic dermatitis adult patients on dupilumab. Although adults experience a lower percentage of AD as compared to children globally, this disease should be addressed to improve the treatment of this condition in adults. The need for the study arises from the fact that there is a dearth of data regarding the efficacy of dupilumab in atopic dermatitis, particularly in the adult population.

## Review

Methodology

Literature Searches

A thorough search was performed on PubMed, Turning Research Into Practice (TRIP), and Cochrane Central Register of Controlled Trials (CENTRAL) databases to look for relevant manuscripts. A combination of keywords and Boolean operators was used for data extraction (Dermatitis, Atopic [Title/Abstract]) OR (dupilumab [Title/Abstract]). The strategy used in the search as well as the total number of articles screened are shown in Table 1.

**Table 1:** Databases searched MeSH: Medical Subject Headings, CENTRAL: Cochrane Central Register of Controlled Trials, TRIP: Turning Research Into Practice

Databases	Boolean operators and MeSH terms	Total number of manuscripts screened
PubMed database	(Dermatitis, Atopic [Title/Abstract]) OR (dupilumab [Title/Abstract]) (filters: English, from 2018 to 2025)	3,602
CENTRAL	(Dermatitis, Atopic [Title/Abstract]) OR (dupilumab [Title/Abstract]) (filters: English, from 2018 to 2025)	2,133
TRIP database	(Dermatitis, Atopic [Title/Abstract]) OR (dupilumab [Title/Abstract]) (filters: English, from 2018 to 2025)	5,827
Total		11,562

Inclusion Criteria

All randomized controlled trials (RCTs) providing information on dupilumab and atopic dermatitis published between 2018 and 2025 were assessed and included in this study. Only RCTs conducted on adults were considered and evaluated. All nine articles were screened independently by three researchers (MK, MR, and SN) in all fields. Full-text articles were included and assessed for eligibility in this systematic review. All RCTs available in the English language were included in the study.

Exclusion Criteria

All non-RCTs, cohort studies, case-control studies, cross-sectional studies, case series, case reports, in vitro studies, and animal experiments were excluded from this systematic review. All commentaries, letters to the editor, expert opinion, and review articles were omitted from this systematic review. All RCTs related to infantile dermatitis were also excluded.

Data Extraction

Data extraction was conducted on the above titles found on the various databases. Each title was initially assessed based on its abstract and then further evaluated by reviewing the full texts. Only those full-text articles that met the eligibility criteria as described above were selected and then proceeded further to make a data synthesis table. The literature evaluation was done separately by MK, MR, and SN. The extracted data included the author, study year, study design, sample size, intervention, dupilumab group, placebo group, patient age group, dupilumab dosing, treatment duration, follow-up period, outcomes evaluated, eczema and severity index, peak pruritus numerical rating scale (NRS), quality of life, potential limitations, and study conclusion.

Risk of Bias Assessment

The Cochrane risk of bias tool for randomized trials (RoB 2) was used for risk of bias assessment. The RoB 2 tool is best suited and implemented to assess the methodological quality of randomized controlled trials. MR independently made the quality assessment stemming from five domains: bias from the randomization process, bias due to deviations from the intended intervention, bias due to missing outcome, bias in the measurement of the outcome, and bias in the selection of the selected result. Data were transferred into the Robvis visualization tool for the development of traffic light plots and weighted bar plots for risk of bias summary and figure.

Results

**Figure 1:**
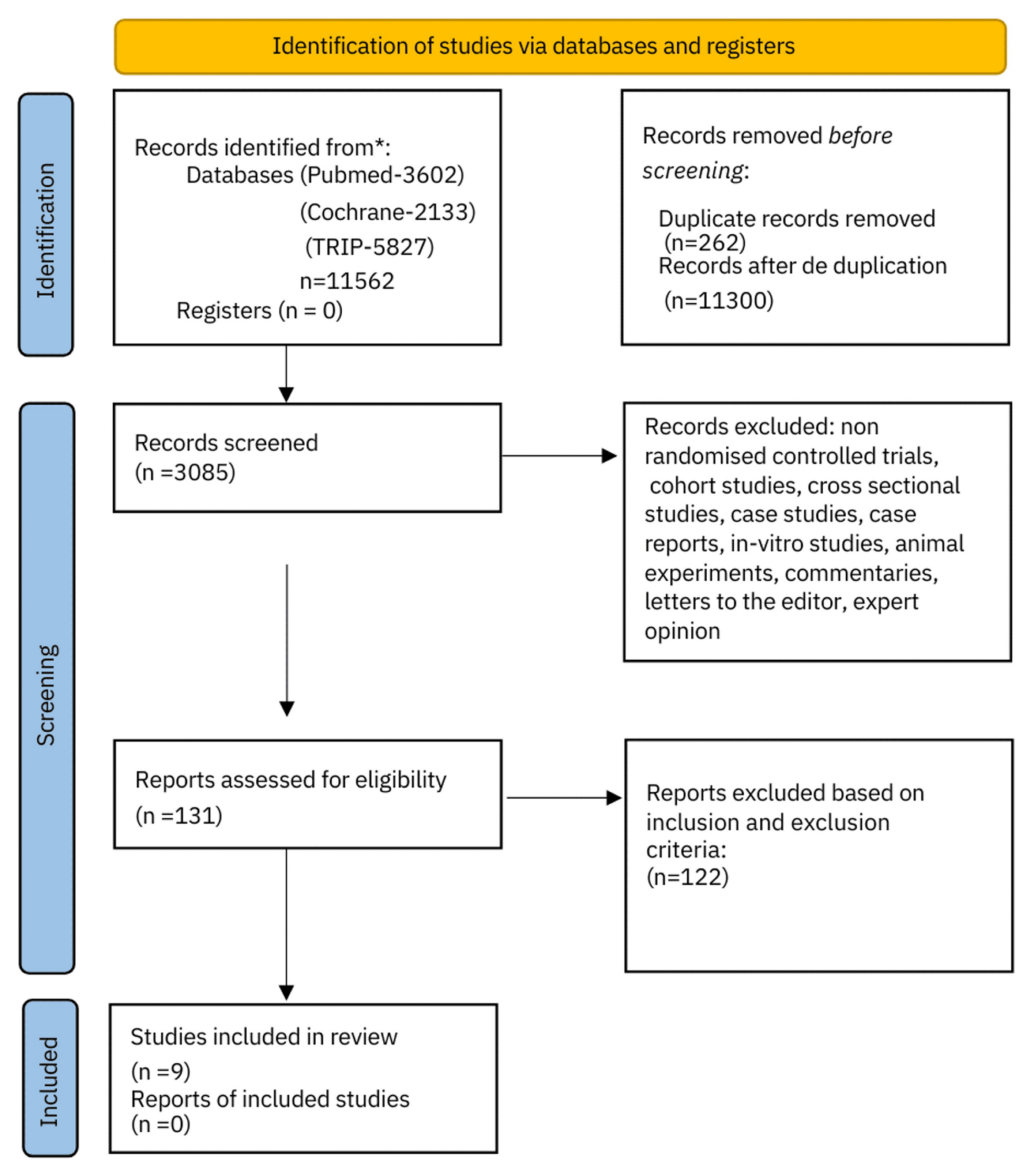
PRISMA 2020 flowchart PRISMA: Preferred Reporting Items for Systematic Reviews and Meta-Analyses, TRIP: Turning Research Into Practice

The literature search generated a total of 11,562 articles (PubMed: 3,602, CENTRAL: 2,133, TRIP: 5,827). Among these, 262 were noted as duplicates and were excluded from the initial analysis. Thus, 11,300 manuscripts were screened after deduplication. Non-RCTs, cohort studies, cross-sectional studies, case-control studies, case series, case reports, in vitro studies, animal experiments, commentaries, letters to the editor, and expert opinions were additionally excluded (n=8,215). A total of 131 full-text articles were assessed for eligibility. An in-depth evaluation and analysis further excluded 122 from the analysis quality assessment as these were not related to adults suffering from atopic dermatitis and receiving dupilumab only as a treatment option. Nine RCTs were finally assessed regarding the clinical efficacy of dupilumab and the quality of life of atopic dermatitis adult patients on dupilumab; these studies were hence included in the systematic review for qualitative synthesis (Figure 1).

**Figure 2:**
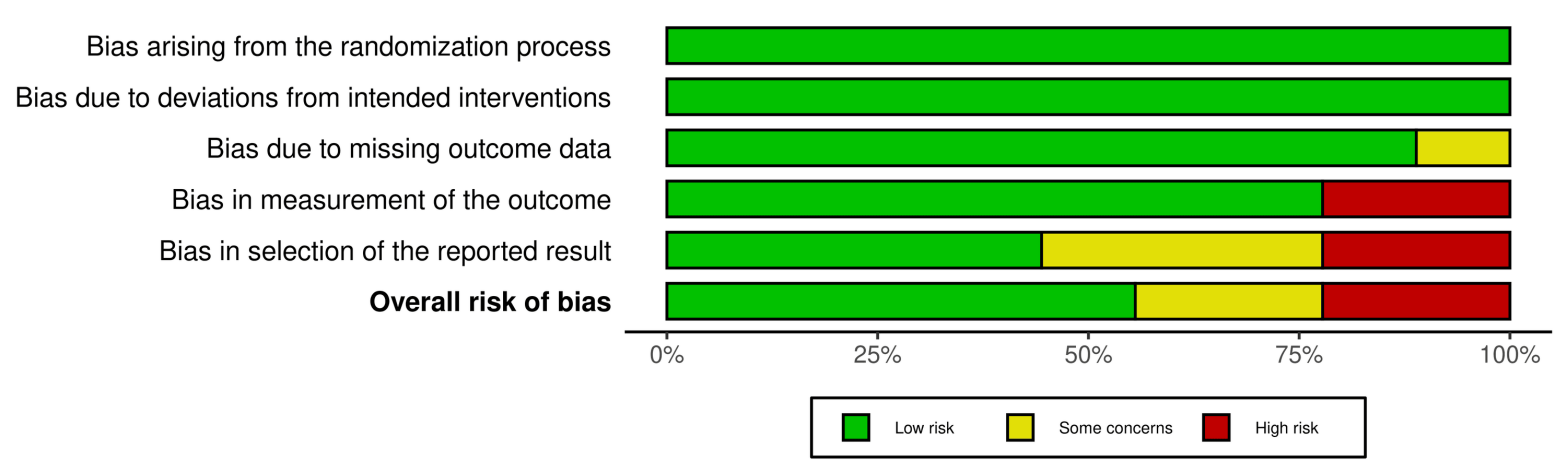
Weighted bar plots showing the summary of risk of bias for the RCTs based on five domains RCTs: randomized controlled trials

**Figure 3:**
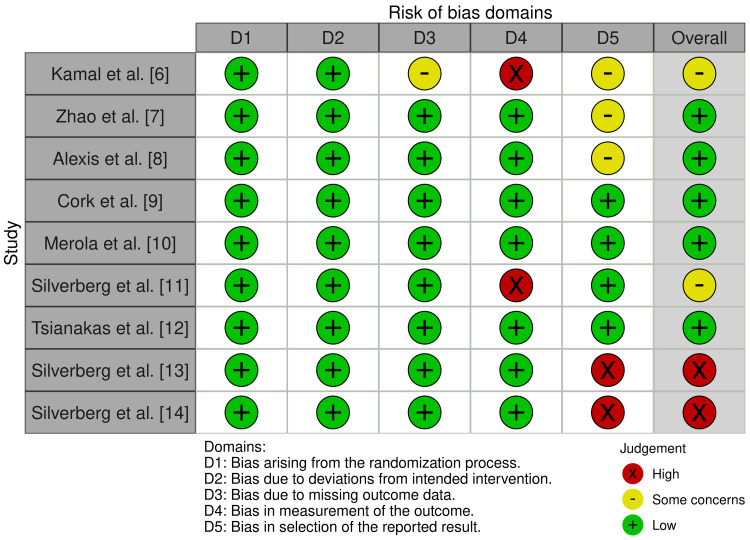
Traffic light plot showing the risk of bias of the nine RCTs included in the final review RCTs: randomized controlled trials

All included RCTs underwent quality assessment using the RoB 2 tool. The results were as follows: randomization process: low risk, 100%; deviations from intended interventions: low risk, 100%; missing outcome data: low risk, 88.9% and some concerns, 11.1%; measurement of outcome: low risk, 77.8% and high risk, 22.2%; and selection of reported result: low risk, 44.4%, some concerns, 33.4%, and high risk, 22.2%. The results for the overall risk of bias for the five domains were as follows: low risk, 55.6%; some concerns, 22.2%; and high risk, 22.2%, which signified some concerns (Figure 2 and Figure 3).

The author, study design, sample size, dupilumab dosing, treatment duration, follow-up period and outcomes, EASI score, NRS, quality of life, limitations, and conclusion of the various selected RCTs are depicted in Tables 2-4.

**Table 2:** Author, study design, sample size, dupilumab group, placebo group, and patient age RCTs: randomized controlled trial

Author, study year	Study design	Sample size	Intervention	Dupilumab group	Placebo group	Patient age group
Kamal et al. [6]	RCT	437	Dupilumab and placebo	368	69	Between 19 and 52 years
Zhao et al. [7]	RCT	165	Dupilumab and placebo	82	83	Between 20 and 36 years
Alexis et al. [8]	RCT	2,058	Dupilumab and placebo	1,304	754	Between 23 and 53 years
Cork et al. [9]	RCT	1,379	Dupilumab and placebo	919	460	Between 22 and 53 years
Merola et al. [10]	RCT	188	Dupilumab and placebo	127	61	Between 18 and 52 years
Silverberg et al. [11]	RCT	2,932	Dupilumab and placebo	1,841	1,091	Between 24 and 52 years
Tsianakas et al. [12]	RCT	64	Dupilumab and placebo	32	32	Between 34 and 44 years
Silverberg et al. [13]	RCT	892	Dupilumab and placebo	449	443	Greater and equal to 18 years
Silverberg et al. [14]	RCT	421	Dupilumab and placebo	106	315	Between 23 and 53 years

**Table 3:** Author, dupilumab dosing, treatment duration, follow-up period, and outcomes evaluated Qw: once a week, q2w: twice a week, EASI: Eczema and Severity Index, NRS: numerical rating scale, SCORAD: Scoring Atopic Dermatitis, POEM: Patient-Oriented Eczema Measure, DLQI: Dermatological Life Quality Index, HADS: Hospital Anxiety and Depression Scale, QoLIAD: Quality of Life index of Atopic Dermatitis, ADRs: adverse drug reactions

Author, study year	Dupilumab dosing	Treatment duration	Follow-up period	Outcomes evaluated
Kamal et al. [6]	200 mg q2w, 300 mg q2w, and 300 mg qw	32 weeks	16 weeks	EASI score and peak pruritus NRS
Zhao et al. [7]	300 mg qw	16 weeks	12 weeks	EASI score, peak pruritus NRS, POEM, DLQI, and ADRs
Alexis et al. [8]	300 mg qw and 300 mg q2w	52 weeks	16 weeks	EASI score, peak pruritus NRS, POEM, DLQI, and ADRs
Cork et al. [9]	300 mg qw and 300 mg q2w	16 weeks	16 weeks	Peak pruritus NRS, SCORAD, DLQI, POEM, HADS, and EQ-5D
Merola et al. [10]	300 mg q2w	24 weeks	12 weeks	Sleep NRS, peak pruritus NRS, SCORAD, POEM, DLQI, and ADRs
Silverberg et al. [11]	300 mg q2w and 300 mg qw	52 weeks	16 weeks	Hospitalization rate and length of AD-related hospitalizations
Tsianakas et al. [12]	300 mg qw	12 weeks	12 weeks	QoLIAD, EASI, SCORAD, peak pruritus NRS, and ADRs
Silverberg et al. [13]	300 mg q2w	32 weeks	16 weeks	EASI, peak pruritus NRS, POEM, and DLQI
Silverberg et al. [14]	300 mg qw	52 weeks	2 months	EASI, DLQI, and ADRs

**Table 4:** Author, EASI, NRS, quality of life, limitations, and study conclusion Qw: once a week, q2w: twice a week, EASI: Eczema and Severity Index, NRS: numerical rating scale, SCORAD: Scoring Atopic Dermatitis, POEM: Patient-Oriented Eczema Measure, DLQI: Dermatological Life Quality Index, HADS: Hospital Anxiety and Depression Scale, QoLIAD: Quality of Life index of Atopic Dermatitis, ADRs: adverse drug reactions, SD: standard deviation, BMI: body mass index, CI: confidence interval, AD: atopic dermatitis, HRQoL: health-related quality of life, IGA: Investigator's Global Assessment, LS: least square, MCID: minimal clinically important difference

Author, study year	EASI	Peak pruritus NRS	Quality of life	Potential limitations	Study conclusion
Kamal et al. [6]	The overall score for placebo was 32.9 (SD: 13.8), whereas the overall score for dupilumab (300 mg q2w) was 33.8 (SD: 14.5; P<0.0001).	The overall score for placebo was 6.3 (SD: 1.8), whereas the overall score for dupilumab (300 mg q2w) was 6.7 (SD: 2.1; P<0.0001).	No adverse effects were observed.	The sample size was small, and race, EASI, and BMI did not follow the statistical inclusion or exclusion criteria.	EASI score and NRS score were higher in dupilumab than in placebo.
Zhao et al. [7]	70.7% of patients receiving dupilumab achieved EASI 50 compared to 28.9% receiving placebo at week 16 (difference: 41.8%; 95% CI: 27.96-55.68; P<0.001).	At week 16, a higher proportion of patients in the dupilumab group (52.4%) than in the placebo group (9.6%) had ≥3 point (95% CI: 30.26-55.34; P<0.001) and ≥4 point (39.0% versus 4.8%; 95% CI: 22.69-45.72; P<0.001) reductions in the weekly average of peak daily pruritus NRS scores (P<0.001 for both endpoints).	Greater reductions were observed in DLQI and POEM scores from baseline. Upper respiratory tract infections, conjunctivitis, allergic conjunctivitis, and injection site reactions were more common with dupilumab.	The treatment period was too short. Only Chinese and Asian populations were included in the study.	EASI and NRS scores were higher in the dupilumab group than in the placebo group. DLQI and POEM scores were less in the dupilumab group.
Alexis et al. [8]	In the Caucasian, Asian, and African subgroups, both dupilumab regimens significantly improved EASI scores.	Only dupilumab (300 mg qw) showed significant improvement versus placebo in mean percent change in peak pruritus NRS.	Dupilumab significantly improved symptoms (POEM), pain/discomfort, and QOL (DLQI). Conjunctivitis and injection site reactions were observed.	The sample size was small. There were variations in mean body weight among racial subgroups. Racial subgroups were self-reported.	Dupilumab has significantly improved EASI, NRS, POEM, and QOL compared to placebo.
Cork et al. [9]	EASI total score was 34.0 (14.4) for placebo, 32.4 (13.3) for dupilumab (300 mg q2w), and 32.5 (13.3) for dupilumab (300 mg qw; P<0.0001).	At week 16, more patients in the dupilumab q2w and dupilumab qw groups had a >3 point improvement in peak pruritus NRS score (48.8% and 50.3%), respectively, versus 15.0% of placebo recipients (P<0.0001).	A greater percentage of patients receiving dupilumab reported no pain, absence of sleep disturbances, improved symptoms of depression and anxiety, and better QOL.	The pooled analysis was not pre-specified.	Dupilumab has significantly improved NRS scores and promotes a better quality of life.
Merola et al. [10]	The LS mean change from baseline to week 12 was -74.1 (38.0) in the dupilumab group compared to -50.3 (38.3) in the placebo group (95% CI: -25.1 (-37.7 to -12.5); P<0.001).	A ≥4 point improvement in peak pruritus NRS was achieved by 74.0% of patients in the dupilumab group compared with 49.2% of patients in the placebo group (95% CI: -38.0 to -17.8; P<0.001).	The mean change in DLQI from baseline was -11.8 (SD 6.5) and -7.5 (SD 6.8) in the dupilumab and placebo groups, respectively (95% CI: -6.4 to -2.6); P<0.001). Conjunctivitis was reported more in patients receiving dupilumab (9.4%).	Wrist-worn actigraph may have caused discomfort in patients with local lesions, resulting in additional pruritus and scratching. No validation data was available for sleep NRS.	Dupilumab has significantly improved the quality and perception of sleep continuity, itch, metrics of AD severity, and QOL in adults with moderate to severe AD.
Silverberg et al. [11]	Not evaluated	Not evaluated	Patients who received dupilumab had lower rates of all-cause hospitalizations when compared to control (3.8 versus 9.0 events per 100 PY) (95% CI: 0.23-0.62; P<0.001; 62% risk reduction).	External generalization of the results was done. The study was dependent on the local healthcare systems.	A lower rate and lower probability of both all-cause and AD-related hospitalizations were observed with dupilumab.
Tsianakas et al. [12]	Dupilumab has resulted in a decrease from baseline (20.6±1.97) in EASI score at week 12 than that observed with placebo (6.3±2.04) (95% CI: -43.86 to -11.80; P=0.001).	The mean LS percentage change from the baseline average weekly pruritus NRS score at week 12 was -50.5±9.27 (95% CI: -69.02 to -31.89; P<0.001).	A significant gain in HRQoL was achieved as the mean change from baseline at week 12 ± SE exceeded the MCID of 2-3 in the dupilumab group. The most common ADRs observed in the dupilumab group were nasopharyngitis, headache, and fatigue.	The QoLIAD was assessed in a small subset of the overall study population. 2. The questionnaire was available in a limited number of different languages.	Dupilumab is effective and has a safety profile for many patients with atopic dermatitis.
Silverberg et al. [13]	The LS mean percentage change in EASI total score from baseline was significantly greater with dupilumab (-48.9%) than with placebo (-11.3%) at week 16, with an overall EASI change from baseline of -17.5 versus -4.3 (P<0.001).	The LS mean percentage change in pruritus NRS was significantly greater with dupilumab (-35.2%) than with placebo (-9.1%) (P<0.001).	Percentage of patients receiving dupilumab versus placebo at week 16: POEM score ≥ 4 point improvement: 57.4% versus 21.0% (P<0.001) and DLQI score ≥ 4 point improvement: 59.3% versus 24.4% (P<0.001).	Week 16 was too early for optimal IGA assessment. The sample size for each subgroup was small.	Dupilumab has significantly improved the EASI total score, pruritus NRS, POEM score, and DLQI score.
Silverberg et al. [14]	At six months, 80.2% of dupilumab-treated versus 40.0% of placebo patients achieved improvement in EASI ≤ 7 (P<0.0001).	At six months, 80.2% of dupilumab-treated versus 40.4% of placebo patients achieved a pruritus NRS score of ≤4 (P<0.0001).	44.3% of dupilumab-treated versus 10.2% of placebo patients achieved a DLQI score of ≤5 at six months (P<0.0001).	Many of the outcomes did not follow the prespecified analyses.	Dupilumab had a satisfactory treatment in all three domains (EASI, pruritus NRS, and DLQI).

Discussion

Dupilumab, a monoclonal antibody, is useful in treating atopic dermatitis (AD). Many different studies have assessed the efficacy, safety, impact on QOL, pharmacokinetics (PK), and pharmacodynamics (PD) of dupilumab in different groups and ethnicities of adults suffering from AD. Most studies have compared the use of dupilumab with a placebo group, whereby the Investigator's Global Assessment (IGA) score, EASI score, NRS score, and POEM score were measured. This systematic review assessed the impact of using dupilumab in treating AD and the adverse effects that could occur during or after the treatment.

Kamal et al. (2022) conducted an RCT with 437 people suffering from AD (moderate to severe). Fifty-eight adults were Japanese, and 379 others were non-Japanese. The patients were randomly assigned into different groups to receive subcutaneous (SC) dupilumab. The groups were 300 mg qw, 300 mg q2w, 200 mg q2w, 300 mg q4w, 100 mg q4w, or placebo for a period of 16 weeks, after which there was a follow-up period of 16 weeks. In general, the numerical data obtained from the Japanese group was greater than that of the non-Japanese group, except for results concerning the trough concentrations (Ctrough) for the 300 mg q2w, where they were similar. The efficacy of dupilumab was found to be dose-dependent and decreased during the follow-up period after treatment. Out of all the groups, the 300 mg qw and q2w were kept for phase III assessment. For the overall population, the EASI score was 32.9 (13.8) in the placebo group, and for the dupilumab 300 mg q2w group, the EASI score was 33.8 (14.5). The NRS score for the placebo overall group was 6.3 (1.8), and for the dupilumab 300 mg q2w group, the NRS score was 6.7 (2.1) [6].

A study conducted by Beck et al. (2022) on 2,827 adults with moderate to severe AD showed similar findings. The patients switched from dupilumab 300 mg qw to 300 mg q2w. The number of patients who achieved an IGA score of less than 1/0 was more after switching to dupilumab 300 mg q2w (53.8% at week 52 versus 64.4% at week 204). The mean EASI score was 3.15 at week 52, which improved to 2.46 at week 204. Treatment-emergent adverse events (TEAEs) were measured to determine the safety, and a decrease was observed (84.9% versus 83.5%). Overall, in both studies by Kamal et al. [6] and Beck et al. [15], dupilumab 300 mg q2w was found to be more efficacious.

Zhao et al. (2021) conducted an RCT on 165 people who suffered from moderate to severe AD. The study included only Asians, and they were divided into two groups (SC dupilumab 300 mg q2w and placebo group) for 16 weeks with a follow-up period of 12 weeks. Of these two groups, 82 received dupilumab, while 83 received a placebo. It was observed that the IGA score was much lower in the dupilumab group than in the placebo (26.8% versus 4.8%). EASI 50 and EASI 90 in dupilumab and placebo were 70.7% versus 28.9% and 40.2% versus 60%, respectively. The reduction in the NRS score for the dupilumab group was greater compared to the placebo group (>3 points: 52.4% versus 9.6%, >4 points: 39% versus 4.8%). Concerning DLQI and POEM scores, both showed a decrease from baseline. TEAEs for dupilumab include upper respiratory tract infections, conjunctivitis, and injection site reactions [7]. A similar study was conducted by Wang et al. (2024) on 575 adult Asians and showed similar results. EASI 50 and EASI 75 achieved by patients on week 16 were 72.58% and 42.72%, respectively. The NRS score was 34.37 at week 16 versus 46.02 at week 52, and DLQI showed a reduction from baseline [16].

Alexis et al. (2019) conducted an RCT with 2,058 patients, among which 1,429 were Caucasians, 501 were Asians, and 128 were African Americans. These three groups were further divided into subgroups, namely, placebo, dupilumab 300 mg qw, and dupilumab 300 mg q2w. There was a significant improvement in EASI for the dupilumab group. The Caucasian and Asian group on dupilumab 300 mg qw showed better results than the placebo group (-25.47 versus -14.91 and -25.46 versus -10.97m respectively), while the African American group on dupilumab 300 mg q2w showed better results than the placebo group (-20.02 versus -11.88). Concerning the pruritus NRS score, the Caucasian and African American group showed better improvement with dupilumab qw versus placebo (-4.24 versus -2.29 and -3.95 versus -2.18), while the Asian group showed better improvement with dupilumab q2w compared to the placebo group (-3.68 versus -1.41). In Caucasians and Asians, DLQI was better in those on dupilumab qw than those on placebo (-10.56 versus -5.52 and -8.24 versus -3.58, respectively). All three groups on dupilumab qw showed better numerical values than those on placebo (Caucasians: -12.88 versus -5.75, Asians: -10.01 versus -1.66, African-Americans: -12.85 versus -6.37). Overall, both groups of dupilumab showed improvement in the QOL of patients compared to those on placebo [8].

Cork et al. (2019) conducted an RCT on 1,379 adults suffering from moderate to severe AD. The adults received either SC dupilumab 300 mg qw or q2w or placebo. The EASI score was slightly higher in those on dupilumab 300 mg q2w than qw. Both dupilumab groups showed improvement in the NRS score compared to the placebo group (q2w: 48.8%, qw: 50.3%, placebo: 15%). There were more patients who achieved an IGA score of 1 or 0 by week 16 in the dupilumab groups compared to the placebo group. It was also found that the dupilumab group experienced no pain, good quality of sleep, and improvement in stress and depression, and they had a better quality of life by week 16 [9]. A similar study was conducted by Silverberg et al. (2020) with a sample size of 1,505 patients. This study also found improvement in pruritus in patients treated with dupilumab 300 mg q2w compared to placebo. Significant improvement was also observed in the POEM score in the dupilumab group. Due to the great improvement of pruritus, the QOL of the patients also improved [11]. Another study by Silverberg et al. (2022) on 2,932 patients found that adults receiving dupilumab had a lower rate of atopic dermatitis hospitalization (a decrease of 60%) [17].

Merola et al. (2023) conducted an RCT on 188 patients divided into two groups: dupilumab 300 mg q2w and placebo for 12 weeks. The EASI score for the dupilumab group showed a percentage change of -74.1 (38) from baseline compared to placebo with -50.3 (38.3). Of the patients in the dupilumab group, 74% showed improvement in the NRS score compared to 49.2% in the placebo. The percentage change in DLQI and POEM scores for dupilumab q2w versus placebo was 4.5 (5.2) versus 9.3 (5.3) and 9.5 (7.1) versus 17.9 (7.4), respectively. There was significant improvement in QOL, quality of sleep, and pruritus in the dupilumab group [10]. Tsianakas et al. (2018) conducted an RCT with a sample size of 64 and discovered that by week 12, the EASI score of the dupilumab group showed a reduction of 20.6±1.97 from the baseline, which was higher than that of the placebo group. The NRS score and HRQoL both showed significant improvement [12].

Silverberg et al. (2019) conducted an RCT on 892 patients and recorded results on week 16. The EASI score (change from baseline) was higher in the dupilumab group than in the placebo group (-48.9% versus -11.3%). The change in NRS score between the two groups showed drastic differences in numerical value (dupilumab group: -35.2%, placebo: -9.1%). The POEM and DLQI scores for dupilumab were higher than those for the placebo group (57.4% versus 21.0% and 59.3% versus 24.4%, respectively) [13]. Silverberg et al. (2021) conducted another RCT on 421 patients who received either topical corticosteroids (TCS) + placebo or dupilumab 300 mg q2w + TCS for 52 weeks. Results were determined after six months. Significant improvement in EASI score was observed in the dupilumab group compared to the placebo group (80.2% versus 40%). NRS score and DLQI both showed better results in the dupilumab group than in the placebo group (80.2% versus 40.4% and 44.3% versus 10.2%, respectively) [14].

There were several limitations in the various studies. Some of the included studies had a small sample size, where it might have been difficult to draw a valid conclusion. In some other RCTs, factors such as race and ethnicity, age, BMI, and weight were not kept constant, so whether these factors affected the results should be investigated. Also, the use of dupilumab was found to cause adverse effects such as upper respiratory tract infections, allergic conjunctivitis, conjunctivitis, injection site reactions, nasopharyngitis, headache, and fatigue in some patients [18-20].

## Conclusions

Both doses of dupilumab 300 mg q2w and 300 mg were successful in improving the symptoms of pruritus, pain, and depression, as well as preventing the spread of the disease in adults with atopic dermatitis. The QOL improved significantly, and the rate of atopic dermatitis hospitalization decreased after the use of dupilumab. It is, however, important to note that the use of dupilumab can cause adverse effects such as conjunctivitis and injection site reactions. Dupilumab is an effective treatment option for adults suffering from atopic dermatitis, but a stringent follow-up period should be advised with patients being sensitized to the possibility of the potential adverse effects/reactions that might occur. These reactions must be swiftly reported to and treated accordingly by the concerned healthcare practitioner.
